# Occurrence and Antimicrobial Resistance Traits of *Escherichia coli* from Wild Birds and Rodents in Singapore

**DOI:** 10.3390/ijerph17155606

**Published:** 2020-08-03

**Authors:** Kar Hui Ong, Wei Ching Khor, Jing Yi Quek, Zi Xi Low, Sathish Arivalan, Mahathir Humaidi, Cliff Chua, Kelyn L. G. Seow, Siyao Guo, Moon Y. F. Tay, Joergen Schlundt, Lee Ching Ng, Kyaw Thu Aung

**Affiliations:** 1 Environmental Health Institute, National Environment Agency, Singapore 138667, Singapore; ong_kar_hui@sfa.gov.sg (K.H.O.); jingyiqjy@hotmail.com (J.Y.Q.); lowzixi@gmail.com (Z.X.L.); sathish_arivalan@nea.gov.sg (S.A.); mahathir_humaidi@nea.gov.sg (M.H.); cliff_chua@nea.gov.sg (C.C.); aung_kyaw_thu@sfa.gov.sg (K.T.A.); 2 National Centre for Food Science, Singapore Food Agency, Singapore 608550, Singapore; khor_wei_ching@sfa.gov.sg; 3 School of Chemical and Biomedical Engineering, Nanyang Technological University, Singapore 637459, Singapore; kelyn.s86@gmail.com (K.L.G.S.); guosiyao@outlook.com (S.G.); moon.tay@ntu.edu.sg (M.Y.F.T.); jschlundt@ntu.edu.sg (J.S.); 4 Nanyang Technological University Food Technology Centre (NAFTEC), Singapore 637459, Singapore; 5 School of Biological Sciences, Nanyang Technological University, Singapore 637551, Singapore

**Keywords:** *Escherichia coli* (*E. coli*), wild birds, rodents, multi-drug resistant (MDR), resistance genes, antimicrobial susceptibility testing, whole genome sequencing

## Abstract

Antimicrobial resistance (AMR) in *Escherichia coli* (*E. coli*) poses a public health concern worldwide. Wild birds and rodents, due to their mobility, are potential vehicles for transmission of AMR bacteria to humans. Ninety-six wild birds’ faecal samples and 135 rodents’ droppings samples were collected and analysed in 2017. Forty-six *E. coli* isolates from wild birds and rodents were subjected to AMR phenotypic and genotypic characterisation. The proportion of *E. coli* isolates resistant to at least one of the antimicrobials tested from wild birds (80.8%) was significantly higher than that of isolates from rodents (40.0%). The proportion of *E. coli* isolates resistant to each antimicrobial class for wild birds was 3.8% to 73.1% and that for rodents was 5.0% to 35.0%. Six out of 26 *E. coli* isolates from wild birds (23.1%) and two out of 20 (10.0%) isolates from rodents were multi-drug resistant (MDR) strains. These MDR *E. coli* isolates were detected with various antimicrobial resistance genes such as *bla_TEM-1B_* and *qnrS1* and could be considered as part of the environmental resistome. Findings in this study suggested that wild birds and rodents could play a role in disseminating antimicrobial resistant *E. coli*, and this underscores the necessity of environment management and close monitoring on AMR bacteria in wild birds and rodents to prevent spreading of resistant organisms to other wildlife animals and humans.

## 1. Introduction

*Escherichia coli* (*E. coli*) is a commensal bacterium found in the guts of animals [1,2]. It can be pathogenic and cause gastroenteritis, bacteraemia and urinary tract infections [2,3,4]. The bacterium is known to be susceptible to selection pressure [2] and has the high competency to pick up and transfer antibiotic resistance genes to and from other bacterial strains [2,5]. Resistance to antimicrobials in clinical and veterinary medicine has been increasingly reported in *E. coli* and this has become a public health concern worldwide [4]. The World Health Organization (WHO) has categorized *Enterobacteriaceae* (including *E. coli*) as pathogens of critical priority for antimicrobial resistance (AMR) investigation [6]. AMR in *E. coli* has been investigated in clinical settings and retail cooked food in Singapore [7,8]. However, to our knowledge, there is limited information on AMR in the environment such as in wild birds and rodents in Singapore. Wild birds and rodents, due to their potential interactions with humans and mobility [9], are potential reservoirs and vectors for transmission of antimicrobial resistant bacteria to humans, through pathways such as contact of faecal materials and contamination of food items [4,9,10]. This study aimed to investigate the occurrence and AMR of *E. coli* from wild birds and rodents in Singapore. The data from the study would enhance our understanding of AMR transmission in the environment and allow subsequent mitigation measures.

## 2. Materials and Methods

### 2.1. Sample Collection, Isolation and Identification

Sample collection: A total of 96 faecal samples from wild birds and 135 samples of rodents’ droppings were conveniently collected in 2017, as part of zoonotic disease surveillance programmes. The wild birds were collected by the Environmental Health Institute of National Environment Agency, where the wild birds’ carcasses were collected from urban areas and recreational parks. An approximate 1 g of wild bird faecal matter was collected after dissection of each wild bird. Samples of rodents’ droppings (rodent species were unidentified) were collected by Rodent Control Unit, of National Environment Agency Central Regional Office. These rodents’ droppings were found at bin chute, drain and kitchen areas.

Sample isolation was carried out according to the method as follows: 1–10 g of wild bird faecal or rodent droppings samples were incubated in 9 mL of Universal Pre-Enrichment Broth (Acumedia, Lansing, MI, USA) under aerobic conditions at 37 ± 1 °C for 16–18 h. A 10 μL loopful of enriched broth was streaked onto Eosine Methylene Blue agar (Acumedia, Lansing, MI, USA) and incubated under the same conditions. Single colonies were streaked on MacConkey agar (Acumedia, Lansing, MI, USA) and incubated under the same conditions for further isolation. The purified colonies were then streaked onto Tryptic Soy Agar (Acumedia, Lansing, MI, USA) and incubated under the same condition. *E. coli* confirmation was performed with an indole test. A pure colony from Tryptic Soy Agar was inoculated into Peptone water (Acumedia, Lansing, MI, USA) and incubated under the same conditions. Five drops (0.5 mL) of Remel™ Kovacs Indole Reagent (Thermo Scientific, Lenexa, KS, USA) was dispensed to the enriched peptone water. A pink ring interfaced between peptone water and indole reagent was observed for *E. coli* isolate. Next, randomly selected separate *E. coli* isolates (one colony per sample) were stored in Brain Heart Infusion broth with 15% glycerol until further usage.

DNA extraction of the presumptive *E. coli* isolates was carried out using DNeasy Blood & Tissue Kits (Qiagen, Hilden, Germany). Confirmation of *E. coli* isolates was performed by 16S ribosomal RNA polymerase chain reaction using forward primer 27f (5′-AGAGTTTGATCCTGGCTCAG-3′) and reverse primer 1492r (5′-GGTTACCTTGTTACGACTT-3′). Polymerase chain reaction conditions are described as follows [11]: A 50 μL reaction mix consisted of 10 μL of 5x Phusion High-Fidelity Buffer (Thermo Scientific, Vilnius, Lithuania), 1 μL of dNTP mix (1st BASE, Seri Kembangan, Malaysia), 0.5 μL (10 µM) of each primer (Integrated DNA Technologies, Singapore), 0.5 μL of Phusion Hot Start II DNA Polymerase (Thermo Scientific, Vilnius, Lithuania), 5 μL of DNA template and 32.5 μL of molecular grade water was used. PCR was conducted using thermocycler (Applied Biosystems, Waltham, MA, USA) with conditions as follows: initial denaturation at 98 °C for 30 s, 35 cycles consisting of denaturation at 98 °C for 10 s, annealing at 50 °C for 30 s and extension at 72 °C for 30 s and a final extension at 72 °C for 10 min. The amplified fragments were visualised at 2% agarose gel. Those isolates with an expected band size of 1465 bp were sequenced using BigDye^®^ Terminator v3.1 Cycle Sequencing Kit (Applied Biosystems, Waltham, MA, USA). Raw sequences were assembled using BioEdit version 7.2.6.1 software. Assembled reads were uploaded to nucleotide BLAST database (https://blast.ncbi.nlm.nih.gov/Blast.cgi?PAGE_TYPE = BlastSearch) (National Centre for Biotechnology Information, United States) for *E. coli* identification (>90%).

### 2.2. Antimicrobial Susceptibility Testing of E. coli Isolates

Susceptibilities to 12 antimicrobial agents of nine different classes were determined by disk diffusion method and interpreted according to the Clinical and Laboratory Standards Institute guideline 2020 [12]. The antimicrobials agents were: Penicillins (Ampicillin 10 µg), Aminoglycosides (Amikacin 30 µg and Gentamicin 10 µg), Beta-lactam/Beta-lactamase Inhibitor Combinations (Amoxicillin/Clavulanic acid 30 µg), Phenicols (Chloramphenicol 30 µg), Quinolones (Ciprofloxacin 5 µg, Nalidixic acid 30 µg, Norfloxacin 30 µg), Cephalosporins (Ceftriaxone 30 µg), Carbapenems (Meropenem 10 µg), Folate Pathway Synthesis (Sulphamethoxazole/Trimethoprim 25 µg) and Tetracyclines (Tetracycline 30 µg) (Oxoid, Basingstoke, UK). *Escherichia coli* ATCC^®^ 25922 strain was used as the quality control strain. Isolates that expressed resistance or intermediate phenotypes were classified as resistant. Isolates that were resistant to three or more classes of antimicrobials were considered multi-drug resistant (MDR) [13].

### 2.3. Extended Spectrum Beta-Lactamases (ESBL) Testing for E. coli Isolates

Resistance to Ceftriaxone by disk diffusion was confirmed for ESBL production, as previously described [7].

### 2.4. Genotypic Characterisation by Whole Genome Sequencing

Based on phenotypic resistance results, eight MDR *E. coli* isolates (indicated in Table 1) were selected and subjected to genotypic analysis by whole genome sequencing. DNA extraction, library preparation and sequencing were performed as previously described [7]. Raw sequence data was deposited into Genbank under Bioproject accession number PRJNA625931. The raw reads were assembled using SPAdes version 3.11.0, with “-careful, -k auto and -cov-cutoff as off” parameters [14]. The genome data were analysed with reference to the ResFinder 3.1 database (https://cge.cbs.dtu.dk/services/ResFinder/) to identify antimicrobial resistance genes and chromosomal point mutations based on the following parameters: minimum length coverage of 60% and minimum identity of 90% (Centre for Genomic Epidemiology, Denmark) [15].

### 2.5. Statistical Analysis

The 95% confidence intervals of proportions were calculated using http://vassarstats.net/prop1.html. Z-scores for two population proportions were calculated using https://www.socscistatistics.com/tests/ztest/default2.aspx.

## 3. Results

### 3.1. Occurrence of E. coli in Wild Birds and Rodents

Of the 96 wild birds’ faecal samples, *E. coli* was detected in 26 (27.1%) [95% CI: 19.2–36.7%] faecal samples from 12 different types of wild birds, with details shown in Table 2. Of 135 rodent droppings, 20 (14.8%) [95% CI: 9.8–21.8%] tested positive for *E. coli* (Table 2).

### 3.2. Antimicrobial Resistance in E. coli Isolated from Wild Birds and Rodents

Antimicrobial susceptibility test results for all the *E. coli* isolates are shown in Table 1. There were 80.8% (21/26) of *E. coli* isolates from wild birds and 40.0% (8/20) of *E. coli* from rodents being resistant to at least one of the antimicrobials tested in the study (Table 3). The proportion of *E. coli* isolates resistant to at least one of the antimicrobials tested from wild birds (80.8%, Z-score 2.8, *p* < 0.05) was significantly higher than that of isolates from rodents (40.0%) (Table 3).

**Figure 1:**
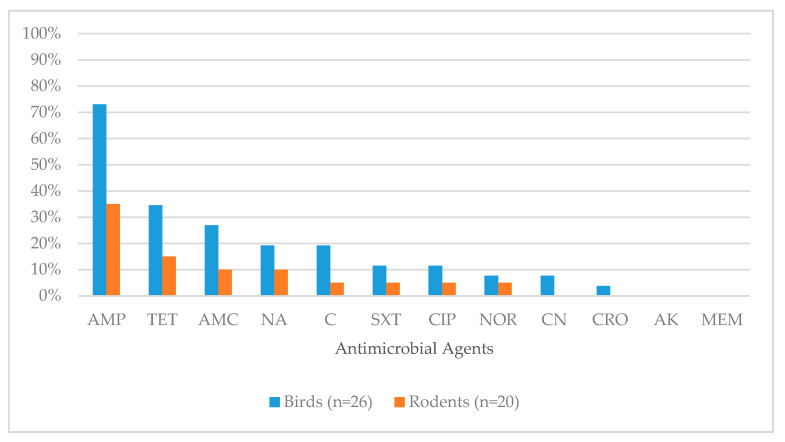
Percentage of antimicrobial resistance in *E. coli* isolates from wild birds and rodents (Corresponding to Table 4). Antimicrobial agents used for testing were AK: Amikacin; AMC: Amoxicillin/Clavulanic acid; AMP: Ampicillin; C: Chloramphenicol; CIP: Ciprofloxacin; CN: Gentamicin; CRO: Ceftriaxone; MEM: Meropenem; NA: Nalidixic acid; NOR: Norfloxacin; SXT: Sulphamethoxazole/Trimethoprim; TE: Tetracycline.

The proportions of *E. coli* isolates resistant to each antimicrobial class for wild birds (3.8% to 73.1%) and rodents (5.0% to 35.0%) are shown in Figure 1 and Table 4.

For isolates from wild birds, the most common phenotypic resistance exhibited was against Penicillins (Ampicillin), followed by Beta-lactam/Beta-lactamase Inhibitor Combinations (Amoxicillin/Clavulanic Acid), Tetracyclines (Tetracycline), Quinolones (Nalidixic acid) and Phenicols (Chloramphenicol). Lower resistance rates (less than or equal to 15.0%) were found for the other remaining five antimicrobial classes (Table 4). No resistance was found for Aminoglycosides (Amikacin) and Carbapenems (Meropenem).

For isolates from rodents, the most common phenotypic resistance exhibited was against Penicillins (Ampicillin). Lower resistance rates (less than or equal to 15.0%) were observed for other remaining seven antimicrobial classes (Table 4). No resistance was found for Aminoglycosides (Gentamicin), Third Generation Cephalosporins (Ceftriaxone), Aminoglycosides (Amikacin) and Carbapenems (Meropenem).

Six out of 26 *E. coli* isolates from wild birds (23.1%) and two out of 20 (10.0%) isolates from rodents were resistant to three or more antimicrobial classes and considered as multi-drug resistant (MDR) strains. One MDR *E. coli* isolate (C1722) recovered from wild birds tested positive for ESBL production.

### 3.3. Distribution of Resistance Genes in Eight MDR E. coli Isolates from Wild Birds and Rodents

At least one antimicrobial resistance gene was detected in all eight MDR *E. coli* isolates from wild birds and rodents (Table 5). An isolate (C1722) from wild bird had 17 resistance genes detected, the highest among the eight MDR *E. coli* isolates. It is worth noting that this isolate was ESBL-producing and harboured *bla_CTM-X-65_* gene and *tet(X)* gene.

Macrolide, Lincosamide and Streptogramin B (MLS) resistance gene (*mdf(A)*) was found in all MDR *E. coli* isolates. An isolate from wild bird (C1776) was detected with additional MLS resistance gene *mph(A)*. Six out of eight (75.0%) MDR *E. coli* isolates were detected with Tetracycline resistance gene (*tet(A)*) while another MDR *E. coli* isolate from wild bird (C1797) was detected with Tetracycline resistance gene *tet(B*). Aminoglycoside resistance genes were detected in 75.0% (6/8) of MDR *E. coli* isolates. The most common Aminoglycoside resistance genes were *aadA1* (37.5%, 3/8), *aadA2* (37.5%, 3/8) and *aph (6)-Id* (37.5%, 3/8). Phenicol resistance genes were detected in 75.0% (6/8) of MDR *E. coli* isolates. *floR* (50.0%, 4/8) was the most frequently detected Phenicol resistance gene, followed by *cmlA1* (37.5%, 3/8) and *catA1* (12.5%, 1/8). ESBL resistance genes were detected in 62.5% (5/8) of the MDR *E. coli* isolates, which comprised of *bla_TEM-1B_* (50.0%, 4/8), *bla_TEM-176_* (12.5%, 1/8) and *bla_CTM-X-65_* (12.5%, 1/8). Plasmid Mediated Quinolone Resistance (PMQR) genes were found in 62.5% (5/8) of MDR *E. coli* isolates, with the common PMQR gene being *qnrS1* (50.0%, 4/8). Another PMQR gene *oqxB* was found in an isolate from wild bird (C1776). Sulphonamide resistance genes were present in 50% (4/8) of MDR *E. coli* isolates, which include *sul3* (50.0%, 4/8) and *sul2* (25.0%, 2/8) resistance genes. Trimethoprim resistance genes (*dfrA12, dfrA14*) were found in 37.5% (3/8) of MDR *E. coli*. Fosfomycin (*fosA3*) resistance gene was detected in one isolate from wild bird (C1722).

Chromosomal point mutations in Quinolone resistant determining regions (QRDR) of *gyrA* and *parC* genes were observed in 37.5% (3/8) of the MDR *E. coli* isolates from wild birds. A *gyrA* mutation for amino acid substitution from Serine to Leucine at 83th position (Ser83Leu) was found in an isolate (C1722), which displayed resistance to Nalidixic Acid and Ciprofloxacin (Table 6). Two isolates (C1776 and C1797) carried double *gyrA* mutations responsible for amino acid change from Serine to Leucine at 83th position (Ser83Leu) and aspartic acid to asparagine at 87th position (Asp87Asn) and *parC* mutation for change from Serine to Isoleucine at 80th position (Ser80Ile). These two isolates were resistant to Nalidixic acid, Ciprofloxacin and Norfloxacin (Table 6).

### 3.4. Comparison between Phenotypic and Genotypic Characteristics of MDR E. coli Isolates

Comparison of antimicrobial phenotype and whole genome sequencing data of MDR *E. coli* isolates (n = 6) from wild birds and rodents (n = 2) was performed for Beta-lactams, Quinolones, Phenicols and Tetracyclines. The remaining five antimicrobial classes were not performed for comparison between antimicrobial resistance phenotype and genotype due to limitations of the antimicrobial susceptibility testing as follows: (1) Macrolides and Fosfomycin were not tested; (2) Many antimicrobial classes of Aminoglycosides (e.g., Kanamycin, Streptomycin) were excluded; (3) A combination of Sulphamethoxazole/Trimethoprim was used and there was no testing for individual antimicrobial classes of Sulphonamides and Trimethoprim.

In general, there was good agreement between phenotypic and genotypic characteristics for Phenicols and Tetracyclines. Discrepancies between phenotypic and genotypic resistance traits were detected in MDR *E. coli* isolates (Table 6). For instance, Quinolones resistance genes were detected in four MDR *E. coli* isolates (C1742, C1758, C1805_1 and 8645_0135) which were, however, phenotypically susceptible to the Quinolones included in this study. Isolate 8655_0114 was phenotypically resistant to Quinolones with no corresponding resistance gene. Three MDR *E. coli* (C1758 and C1797, 8655_0114) were phenotypically resistant to Penicillins and Beta-lactam/Beta-lactamase Inhibitor Combinations but there was no ESBL resistance gene detected.

## 4. Discussion

To our knowledge, this is the first report on the occurrence and antimicrobial resistant phenotype and genotype in *E. coli* isolates from wild birds and rodents in Singapore.

This study revealed that the occurrence of *E. coli* in wild birds (27.1%) in Singapore was relatively lower than that reported in other countries such as Switzerland (53.7%) and Saudi Arabia (93.0%) [16,17]. Similarly, the occurrence of *E. coli* in rodents (14.8%) was relatively lower than it is reported in other countries such as Trinidad and Tobago (83.8%) and Canada (62.7%) [18,19]. One possible reason for relatively lower occurrences on both wild birds and rodents could be due to differences in sampling and laboratory methods used in respective studies (e.g., the convenient collection of samples used in this study) that rendered comparison of occurrence data between studies challenging. Furthermore, the occurrence data could be affected by storage conditions of the collected samples. Another limitation of our study was that we were unable to identify the rodent species despite it being known that common rodent species found in Singapore include Black Rat (*Rattus norvegicus*), Brown Rat (*Rattus rattus*) and House Mouse (*Mus musculus)* [20]. The occurrence of *E. coli* could hypothetically originate from these pools of common rodent species. Our study provided an insight into the occurrence of *E. coli* isolates from wild birds and rodents in Singapore, which enhances our understanding of the local epidemiology of *E. coli* and could guide future epidemiological studies.

The proportion of *E. coli* isolates resistant to at least one of the antimicrobials tested from wild birds was significantly higher than that of isolates from rodents. Although different in behavior, diet and migration potential with regard to species, wild birds generally have a higher movement pattern than rodents and could have higher exposure to antimicrobial resistance determinants in the ecological niches. This could lead to a higher probability for wild birds in disseminating AMR determinants to humans or other wildlife animals [21]. Our results differ from a previous study in Singapore which indicated there was no phenotypic antimicrobial resistance detected in *Salmonella* isolates recovered from wild birds [22]. The difference in phenotypic resistance observed for *E. coli* and *Salmonella* isolates could be due to the fact that *E. coli* has a greater ability to acquire resistance than *Salmonella*, making *E. coli* more susceptible to antimicrobial selection pressure than *Salmonella* for the tested antimicrobials [23,24]. This implies the importance of using multiple bacteria organisms (both commensal and pathogenic) as AMR indicators in surveillances for better understanding of the distribution of resistant organisms or resistance determinants in the environment.

The antimicrobial resistance rate among *E. coli* isolates could be related to the usage of antimicrobials. *E. coli* isolates from both wild birds and rodents were phenotypically resistant to Penicillins, whereas isolates recovered from wild birds also displayed resistance to Beta-lactam/Beta-lactamase Inhibitor Combinations and Tetracyclines. These antimicrobials are commonly used in clinical and agricultural sectors [25,26,27,28,29,30]. This is of public health concern as these antimicrobials are the first-line drugs of choice for empirical treatment of infections caused by *E. coli*. The widespread resistance to these antimicrobials will render these antimicrobials ineffective for the treatment of infections and will increase the need for an alternative antimicrobial therapy option.

The lower percentage (less than or equal to 15.0%) of resistance observed in *E. coli* isolates from wild birds to antimicrobial classes of Aminoglycosides and Third Generation Cephalosporins suggested that there could be a relatively lower selection pressure of these antimicrobial classes as compared to other commonly used antimicrobials in wild birds. As these antimicrobial classes are not commonly used in food animal production [4,31], nor in clinical settings as a first-line drug for *E. coli* infection but as a drug of choice for invasive/resistant infections [4,32,33], the level of antimicrobial residual pollution in the environment is expected to be lower, and consequently lower exposure and selection pressure for wild birds [34]. Nevertheless, further monitoring of efficacy for these antimicrobials remains necessary.

Our study detected *E. coli* isolates from wild birds and rodents that were resistant to Quinolones (Nalidixic Acid), which is an indicator of reduced susceptibility for Fluoroquinolones (e.g., Ciprofloxacin and Norfloxacin). Indeed, Nalidixic Acid resistant *E. coli* isolates from wild birds (n = 5) also showed resistance to Ciprofloxacin (60.0%, 3/5) and Norfloxacin (40.0%, 2/5). A similar observation was found in rodents where Nalidixic Acid-resistant *E. coli* isolates (n = 2) were resistant to Ciprofloxacin (50.0%, 1/2) and Norfloxacin (50.0%, 1/2). These findings indicated the possible increasing trend for Fluroquinolones-resistant organisms present in the environment. It is important to implement continuous monitoring of Quinolone and Fluroquinolone resistance in *E. coli* from wild birds and rodents in order to prevent the spread of the resistance determinants into environmental niches.

All MDR isolates in our study were detected with MLS resistance gene *mdf(A).* A study showed that this resistance gene is expressed constitutively in *E. coli* [35] and it encodes for a multidrug efflux pump [36]. Another study has shown that the expression of *mdf(A)* in *E. coli* would confer multidrug resistance [37]. Isolate C1776 (from wild bird) harboured another MLS resistance gene *mph(A)*, which encodes for enzymes capable of inactivating Erythromycin (a type of Macrolides) [38].

Our observation of *tet(A)* being the most common tetracycline resistance gene in all MDR isolates was in agreement with other studies in birds and rodents [26,39,40]. Isolate C1797 from wild bird was detected with the *tet(B)* gene—another gene which codes for efflux pump to transport Tetracycline out of bacterial cell [7]. Isolate C1722 from wild bird harboured *tet(X)*, which is the first described tetracycline resistance gene that encodes for an enzyme which inactivates Tetracycline. The *tet(X)* gene was previously identified in environmental bacteria from soil, sewage plants and human clinical samples [41].

Aminoglycosides resistance genes encode for enzymes such as acetyltransferases (such as *aac(3)-IV* detected in our study), nucleotidyltransferases (such as *aadA1* and *aadA2* found in this study) or phosphotransferases (such as *aph(3′)-Ia and aph (6)-Id* identified in our study) which inactivate Aminoglycosides. The most common Aminoglycoside genes detected were *aadA1, aadA2* and *aph (6)-Id.* Genes *aadA1* and *aadA2* confer resistance to Aminoglycosides (e.g., Spectinomycin and Streptomycin) [42] and they were also found in a study on birds from Australia [43]. The gene *aph (6)-Id* was reported as the most commonly detected gene in *E. coli* clinical isolates from Egypt [44].

Both Phenicol resistance genes *floR* and *cml1a* that encode for efflux transporter [45] were detected in our study. The *floR* gene was reported in *Klebsiella pneumoniae* from clinical isolates [46] and *E. coli* from cattle [47], while *cml1a* was found in *E. coli* from poultry [48]. Another gene *catA1* was found in isolate C1797, which encodes for an enzyme that inactivates Phenicols [45] and it was previously also reported in birds [43].

Two types of ESBL resistance genes (*bla_TEM_* and *bla_CTX-M_*) were detected in MDR *E. coli* isolates and both resistance genes encode for Amber Class A Beta-lactamases [45]. The most commonly detected ESBL resistance gene *bla_TEM-1B_*, encodes for TEM-1 Beta-lactamase that hydrolyses Penicillins and First Generation Cephalosporins [7], and was reported in another study on birds [49]. The gene *bla_TEM-176_,* was reported in birds such as gull and rook [9]. On the other hand, *bla_CTX-M-65_* which encodes for CTX-M Beta-lactamase that hydrolyses Penicillins and First to Third Generation Cephalosporins [50], was reported in clinical isolates [51] and raw retail chicken [52].

*Qnrs1* encodes for a protein which binds to and protects both DNA gyrase and topoisomerase IV from inhibition by Quinolones [53]. *Qnrs1* represented the most commonly detected PMQR gene among MDR isolates in this study and this is in agreement with other studies on *E. coli* and *Enterobacteriaceae* from birds [54,55]. Another PMQR gene *oqxB* that was detected in this study, was shown to encode for multidrug efflux pump [56]. This gene was found in *Enterobacteriaceae* from rooks throughout the European continent [55] and human clinical isolates from Korea [56]. Sulphonamide resistance genes *sul2* and *sul3* were identified, which is in line with other reports on birds [43,49] and rodents [40].

This study detected three MDR *E. coli* isolates with chromosomal point mutations in QRDR. Mechanisms of resistance to Quinolones include target gene mutations, active efflux pumps, decreased permeability for outer membrane and acquisition of resistance genes such as *qnrS1* [30,57]. Target gene mutations include alteration of QRDR in DNA gyrase subunit A (*gyrA)* and topoisomerase IV subunit C (*parC*) [58]. In this study, detection of *gyrA* and *parC* mutations was found in 37.5% of the MDR *E. coli* isolates. These *gyrA* and *parC* mutations were identical as reported previously for Quinolone-resistant *E. coli* strains [57]. One ESBL-producing and MDR *E. coli* isolate (C1722) which displayed resistance to Nalidixic Acid and Ciprofloxacin was found to have a single *gyrA* mutation. Our finding differed from the report which indicated that a single *gyrA* mutation would result in resistance to Nalidixic Acid and an additional mutation in *gyrA* or *parC* would be required for resistance in Ciprofloxacin [57]. The different phenotypic resistance observed in the C1722 isolate could be due to the resistance mechanism for co-existence of the ESBL resistance gene (such as *bla_CTM-X-65_*) and single *gyrA* mutation, which was reported in a clinical study from China [51].

Our data, supported by other studies, suggest that the antimicrobial resistance genotype does not always correspond well with phenotypic expression and vice versa [59,60]. It is known that there are multiple complex mechanisms that can lead to bacteria becoming resistant to antimicrobials [59,60]. In the absence of corresponding resistance genes that encode for proteins responsible for enzymatic degradation of antimicrobials and alteration of bacterial proteins targeted by antimicrobials, isolates can exhibit resistance due to other mechanisms such as porin loss and efflux pumps [61]. For example, the detection of *mdf(A)* in MDR *E. coli* isolate from rodent (8655_0114), which encodes for a multidrug efflux pump, could possibly explain the observation that this isolate was conferred resistance to many antimicrobials but had no detectable corresponding resistance genes.

In our study, PMQR genes were detected in four MDR *E. coli* isolates (C1742, C1758, C1805_1 and 8645_0135) which were, however, phenotypically susceptible to the Quinolones tested. This is congruent with a study on *E. coli* from environmental samples in pig farms, which reported that PMQR genes alone are insufficient to confer resistance to Quinolones [62]. As discussed in the previous paragraph, further mechanisms such as mutations in QRDR regions would confer resistance to Quinolones. Despite the absence of phenotypic resistance traits, isolates harbouring antimicrobial resistance genes may be subjected to a transfer of resistance determinants to other bacterial isolates/species via horizontal gene transfer [63]. Thus, risks posed by susceptible isolates carrying resistance determinants should not be underestimated. Our findings suggested that the bacterial isolates should be characterised for both phenotypic and genotypic resistance traits, for a holistic interpretation and more thorough risk assessment. To have a more comprehensive antimicrobial resistance gene portfolio of the *E. coli* isolates obtained in this study, further genotypic screening could be carried out for the remaining antimicrobial resistant *E. coli* isolates.

Our study reports antimicrobial resistant *E. coli* isolates from wild birds and rodents in Singapore. In addition, genotypic characterisation by whole genome sequencing revealed the diversity of resistance genes in eight MDR *E. coli* isolates, which demonstrated the value of whole genome sequencing as an epidemiological tool for further understanding of antimicrobial resistance gene profiles in bacterial isolates. Once wild birds and rodents acquire antimicrobial resistant bacteria, these bacteria could continue to colonise and infect the hosts [64]. Therefore, wild birds and rodents could play a role for the dissemination of antimicrobial resistant bacteria and/or genes across different wildlife species and environmental sectors, perhaps via their faecal materials, as supported by other *E. coli* studies on wild birds and rodents [10,16,21,40,65]. An increasing number of cities today are undergoing urban rewilding, which transforms dense urban areas into green cities with nature assimilated. While the increased biodiversity in cities brings many benefits, it could also facilitate the crossovers of antimicrobial resistance pathogens or genes between urban and wildlife ecosystems. Hence, a close monitoring programme on the antimicrobial resistant bacteria in wildlife, especially in those animals that are in close proximity to human habitats, is recommended to complement surveillance systems in food animals, food and humans.

## 5. Conclusions

This study provides baseline data of occurrence and antimicrobial resistance characteristics of *E. coli* in wild birds and rodents representing a part of the environment in Singapore. Wild birds and rodents could play a contributing role to further spread antimicrobial resistance to other wildlife and environmental sectors through faecal contamination. The findings of our study highlight the importance of (i) environment management; (ii) close-monitoring on AMR bacteria, particularly in the potential reservoirs such as wild bird and rodents, and (iii) a deeper understanding of AMR transmission in our environment.

## Figures and Tables

**Table 1:** Antimicrobial susceptibility profiles of *E. coli* isolates from wild birds and rodents (#: Multi-drug resistant strain; S: Susceptible; I: Intermediate; R: Resistant. Antimicrobial agents used for testing were AK: Amikacin; AMC: Amoxicillin/Clavulanic acid; AMP: Ampicillin; C: Chloramphenicol; CIP: Ciprofloxacin; CN: Gentamicin; CRO: Ceftriaxone; MEM: Meropenem; NA: Nalidixic acid; NOR: Norfloxacin; SXT: Sulphamethoxazole/Trimethoprim; TE: Tetracycline).

Sample ID	Sample Type	Source	AK30	AMC30	AMP10	CRO30	C30	CIP5	CN10	MEM10	NA30	NOR10	SXT25	TE30
C1776#	Wild Bird	Crow	S	S	R	S	R	R	S	S	R	R	R	R
C1775	Wild Bird	Myna	S	S	R	S	S	S	S	S	S	S	S	S
8657-0407-0411-1	Rodent	Rodent	S	S	S	S	S	S	S	S	S	S	S	S
8657-0948-0411	Rodent	Rodent	S	S	I	S	S	S	S	S	S	S	S	S
8645-0135#	Rodent	Rodent	S	S	R	S	R	S	S	S	S	S	R	R
C1797#	Wild Bird	Crested Goshawk	S	S	I	S	R	R	S	S	R	R	S	R
C1809-1	Wild Bird	Hooded Pitta	S	S	I	S	S	S	S	S	S	S	S	S
C1789	Wild Bird	Crow	S	S	I	S	S	S	S	S	S	S	S	S
C1798	Wild Bird	Black-naped oriole	S	S	I	S	S	S	S	S	S	S	S	S
HHK-M04-060417-1	Rodent	Rodent	S	S	S	S	S	S	S	S	S	S	S	S
HHK-M04-060417-3	Rodent	Rodent	S	S	I	S	S	S	S	S	S	S	S	S
8642-0159-0410-1	Rodent	Rodent	S	S	S	S	S	S	S	S	S	S	S	S
C1742#	Wild Bird	Yellow Bittern	S	I	R	S	R	S	S	S	S	S	S	R
C1779	Wild Bird	Crow	S	S	S	S	S	S	S	S	S	S	S	S
C1805-1#	Wild Bird	Crow	S	I	R	S	R	S	S	S	S	S	S	R
8657-0146-0224	Rodent	Rodent	S	S	S	S	S	S	S	S	S	S	S	S
NHA-M02-230117	Rodent	Rodent	S	S	S	S	S	S	S	S	S	S	S	S
8657-0258-0411-1	Rodent	Rodent	S	S	S	S	S	S	S	S	S	S	S	S
7574-0224-0224	Rodent	Rodent	S	S	I	S	S	S	S	S	S	S	S	S
C1802-1	Wild Bird	Crow	S	S	S	S	S	S	R	S	S	S	S	R
8646-0251-0301	Rodent	Rodent	S	S	I	S	S	S	S	S	S	S	S	S
HHK-M05-160317	Rodent	Rodent	S	S	S	S	S	S	S	S	S	S	S	S
C1743E	Wild Bird	Black Bittern	S	S	S	S	S	S	S	S	S	S	S	S
C1781	Wild Bird	Black Bittern	S	S	I	S	S	S	S	S	S	S	S	S
HHK-M07-060417-1	Rodent	Rodent	S	I	R	S	S	S	S	S	S	S	S	R
C1806-1	Wild Bird	Pied Fantail	S	S	S	S	S	S	S	S	S	S	S	S
C1736	Wild Bird	Brahmin Kite	S	S	S	S	S	S	S	S	S	S	S	S
8645-0205-0411-1	Rodent	Rodent	S	S	S	S	S	S	S	S	S	S	S	S
C1795	Wild Bird	Sparrow Hawk	S	S	I	S	S	S	S	S	R	S	S	S
C1794	Wild Bird	Grey Heron	S	I	R	S	S	S	S	S	I	S	S	R
NRS-M02-150317	Rodent	Rodent	S	S	S	S	S	S	S	S	S	S	S	S
C1783	Wild Bird	Scops Owl	S	S	S	S	S	S	S	S	S	S	S	S
C1770	Wild Bird	Myna	S	S	S	S	S	S	S	S	S	S	S	R
C1758#	Wild Bird	Black Bittern	S	I	R	S	S	S	S	S	S	S	R	R
C1757	Wild Bird	Yellow Bittern	S	S	I	S	S	S	S	S	S	S	S	S
C1750	Wild Bird	Sparrow Hawk	S	I	R	S	S	S	S	S	S	S	S	S
C1737	Wild Bird	Crow	S	S	I	S	S	S	S	S	S	S	S	S
C1740	Wild Bird	Crow	S	R	R	S	S	S	S	S	S	S	S	S
C1803-2	Wild Bird	Crow	S	S	I	S	S	S	S	S	S	S	S	S
8656-0339-0224	Rodent	Rodent	S	S	S	S	S	S	S	S	S	S	S	S
TAH-M01-210317	Rodent	Rodent	S	S	S	S	S	S	S	S	R	S	S	S
HHK-M04-060417-1A	Rodent	Rodent	S	S	S	S	S	S	S	S	S	S	S	S
8655-0114#	Rodent	Rodent	S	R	R	S	S	R	S	S	R	R	S	R
C1738	Wild Bird	Crow	S	S	I	S	S	S	S	S	S	S	S	S
C1722#	Wild Bird	Crow	S	R	R	R	R	I	R	S	R	S	R	R
HHK-M06-060417-1	Rodent	Rodent	S	S	S	S	S	S	S	S	S	S	S	S

**Table 2:** Occurrence of *E. coli* in wild birds and rodents.

Types of Samples	Percentage of Samples Positive for *E. coli*	Sample Name (Scientific Name)	No. of *E. coli* Isolates
Wild Birds	27.1% (26/96) [95% CI: 19.2–36.7%]	Crow (*Corvus* spp.)	Ten
		Black Bittern (*Ixobrychus flavicollis*)	Three
		Myna (*Acridotheres* spp.)	Two
		Sparrow Hawk (*Accipiter* spp.)	Two
		Yellow Bittern (*Ixobrychus sinensis*)	Two
		Black-naped oriole (*Oriolus chinensis*)	One
		Brahminy Kite (*Haliastur indus*)	One
		Crested Goshawk (*Accipiter trivirgatus*)	One
		Grey Heron (*Ardea cinerea*)	One
		Hooded Pitta (*Pitta sordida*)	One
		Pied Fantail (*Rhipidura javanica*)	One
		Scops Owl (*Otus* spp.)	One
Rodents	14.8% (20/135) [95% CI: 9.8–21.8%]	Black Rat/Brown Rat/House Mouse (*Rattus rattus/Rattus norvegicus/Mus musculus*)	20

**Table 3:** Percentage of *E. coli* isolates from wild birds and rodents resistant to at least one antimicrobial.

Wild Birds	Rodents	Z-Score
80.8% (21/26) [95% CI: 62.1–91.5%]	40.0% (8/20) [95% CI: 21.9–61.3%]	2.8 (*p* < 0.05)

**Table 4:** Percentage of antimicrobial resistance in *E. coli* isolates from wild birds and rodents (Corresponding to Figure 1).

Antimicrobial Class	Antimicrobial Agent Tested in the Study	Percentage of Isolates Showing Resistant Phenotype (n)	
		Wild Birds (26)	Rodents (20)
Penicillins	Ampicillin	73.1% (19/26)	35.0% (7/20)
Tetracyclines	Tetracycline	34.6% (9/26)	15.0% (3/20)
Beta-lactam/beta-lactamase Inhibitor Combinations	Amoxicillin/Clavulanic acid	26.9% (7/26)	10.0% (2/20)
Quinolones	Nalidixic acid (Quinolone)	19.2% (5/26)	10.0% (2/20)
Phenicols	Chloramphenicol	19.2% (5/26)	5.0% (1/20)
Folate Pathway Synthesis	Sulphamethoxazole/Trimethoprim	11.5% (3/26)	5.0% (1/20)
Quinolones	Ciprofloxacin (Fluoroquinolone)	11.5% (3/26)	5.0% (1/20)
Quinolones	Norfloxacin (Fluoroquinolone)	7.7% (2/26)	5.0% (1/20)
Aminoglycosides	Gentamicin	7.7% (2/26)	0.0%
Third Generation Cephalosporins	Ceftriaxone	3.8% (1/26)	0.0%
Aminoglycosides	Amikacin	0.0%	0.0%
Carbapenems	Meropenem	0.0%	0.0%

**Table 5:** Distribution of resistance genes in the eight multi-drug resistant *E. coli* isolates.

Isolate ID	Sample Source	Sample Description	Resistance Genes (n)	Aminoglycoside	ESBL	Quinolone	Fosfomycin	MLS ^a^	Phenicol	Sulphonamide	Tetracycline	Trimethoprim	Chromosomal Point Mutations
C1722	Wild bird	Crow	17	*aac(3)-IV, aadA2, aph(3′)-Ia, aph(3″)-Ib, aph(4)-Ia, aph(6)-Id,*	*bla_CTX-M-65_, bla_TEM-1B_*	*-*	*fosA3*	*mdf(A)*	*cmlA1, floR*	*sul2, sul3*	*tet(A), tet(X)*	*dfrA12*	*gyrA* (Ser83Leu)
C1742	Wild bird	Yellow Bittern	7	*aph(3′)-Ia*	*bla_TEM-176_*	*qnrS1*	*-*	*mdf(A)*	*floR*	*-*	*tet(A)*	*dfrA14*	*-*
C1758	Wild bird	Black Bittern	4	*aph(6)-Id*		*qnrS1*	*-*	*mdf(A)*	*-*	*-*	*tet(A)*	*-*	*-*
C1776	Wild bird	Crow	11	*aadA1, aadA2, aph(3″)-Ib, aph(6)-Id*	*bla_TEM-1B_*	*oqxB*	*-*	*mdf(A), mph(A)*	*cmlA1*	*sul3*	*tet(A)*	*-*	*gyrA,* (Ser83Leu)*, gyrA*(Asp87Asn) & *parC* (Ser80Ile).
C1797	Wild bird	Crested Goshawk	3	*-*	*-*	*-*	*-*	*mdf(A)*	*catA1*	*-*	*tet(B)*	*-*	*gyrA* (Ser83Leu), *gyrA*(Asp87Asn) & *parC* (Ser80Ile).
C1805-1	Wild bird	Crow	7	*aadA1*	*bla_TEM-1B_*	*qnrS1*	*-*	*mdf(A)*	*floR*	*sul3*	*tet(A)*	*-*	*-*
8645-0135	Rodent	Rodent	11	*aadA1, aadA2*	*bla_TEM-1B_*	*qnrS1*	*-*	*mdf(A)*	*cmlA1, floR*	*sul2, sul3*	*tet(A)*	*dfrA12*	*-*
8655-0114	Rodent	Rodent	1	*-*	*-*	*-*	*-*	*mdf(A)*	*-*	*-*	*-*	*-*	*-*

MLS ^a^—Macrolide, Lincosamide and Streptogramin B.

**Table 6:** Comparison of antimicrobial resistance phenotype and genotype of the eight multi-drug resistant *E. coli* isolates for the selected antimicrobial agents (AMC: Amoxicillin/Clavulanic acid; AMP: Ampicillin; C: Chloramphenicol; CIP: Ciprofloxacin; CRO: Ceftriaxone; NA: Nalidixic acid; NOR: Norfloxacin; TE: Tetracycline).

Isolate ID	Sample Source	Sample Description	Beta-lactams		Quinolones		Phenicols		Tetracyclines	
			Phenotype	Genotype	Phenotype	Genotype	Phenotype	Genotype	Phenotype	Genotype
C1722	Wild bird	Crow	AMC *, AMP, CRO	*bla_CTX-M-65,_ bla_TEM-1B_*	CIP, NA	Chromosomal mutations *gyrA* (Ser83Leu)	C	*cmlA1, floR*	TE	*tet(A), tet(X)*
C1742	Wild bird	Yellow Bittern	AMC *, AMP	*bla_TEM-176_*	-	*qnrS1*	C	*floR*	TE	*tet(A)*
C1758	Wild bird	Black Bittern	AMC *, AMP	*-*	-	*qnrS1*	-	*-*	TE	*tet(A)*
C1776	Wild bird	Crow	AMP	*bla_TEM-1B_*	CIP, NA, NOR	*oqxB,* Chromosomal mutations *gyrA* (Ser83Leu) *gyrA* (Asp87Asn) & *parC* (Ser80Ile)	C	*cmlA1*	TE	*tet(A)*
C1797	Wild bird	Crested Goshawk	AMP	*-*	CIP, NA, NOR	Chromosomal mutations *gyrA* (Ser83Leu) *gyrA* (Asp87Asn) & *parC* (Ser80Ile)	C	*catA1*	TE	*tet(B)*
C1805_1	Wild bird	Crow	AMP, AMC *	*bla_TEM-1B_*	-	*qnrS1*	C	*floR*	TE	*tet(A)*
8645_0135	Rodent	Rodent	AMP	*bla_TEM-1B_*	-	*qnrS1*	C	*cmlA1, floR*	TE	*tet(A)*
8655_0114	Rodent	Rodent	AMC *, AMP	-	CIP, NA, NOR	-	-	-	TE	-

* Denotes a combination of two different classes of antimicrobial agents being used in the antimicrobial susceptibility testing.
